# PRMT6 methylation of STAT3 regulates tumor metastasis in breast cancer

**DOI:** 10.1038/s41419-023-06148-6

**Published:** 2023-10-09

**Authors:** Qianzhi Chen, Qingyi Hu, Yan Chen, Na Shen, Ning Zhang, Anshu Li, Lei Li, Junjun Li

**Affiliations:** 1 Department of Breast and Thyroid Surgery, Union Hospital, Tongji Medical College, Huazhong University of Science and Technology, Wuhan, Hubei China; 2 Department of Hematology, Wuhan No. 1 Hospital, 430022 Wuhan, China; 3 Department of Ultrasound, Union Hospital, Tongji Medical College, Huazhong University of Science and Technology, Wuhan, China; 4 Department of Gastrointestinal Surgery, Union Hospital, Tongji Medical College, Huazhong University of Science and Technology, 430022 Wuhan, China; 5 Department of Neurosurgery, Union Hospital, Tongji Medical College, Huazhong University of Science and Technology, Wuhan, China

**Keywords:** Breast cancer, Cell invasion

## Abstract

Overcoming distant metastasis stands as a paramount challenge in enhancing the outcomes of breast cancer treatments. Thus, delving deeper into comprehending the intricate mechanisms underlying breast cancer metastasis becomes imperative, offering potential avenues for pioneering therapeutic approaches. PRMT6, an arginine N-methyltransferase, possesses the ability to methylate both histone and non-histone proteins. It has been reported that methylation of non-histone proteins impacts their cellular localization, stability, and activation, consequently influencing tumor progression. However, the extent to which PRMT6-mediated non-histone protein methylation influences cancer cell metastasis, particularly in the context of breast cancer, remains elusive. In this study, we established that PRMT6 exerted a positive regulatory influence on breast cancer metastasis through both in vivo and in vitro experiments. Mechanistically, we innovatively revealed that PRMT6 asymmetrically di-methylated STAT3 at arginine 729 (STAT3 R729me2a). This modification proved indispensable for STAT3’s membrane localization, its interaction with JAK2, STAT3 Y705 phosphorylation, and PRMT6-driven cancer cell metastasis. From a clinical perspective, we unearthed the promising potential of STAT3 R729me2a as a robust prognostic marker for predicting the overall survival time of breast cancer patients. In terms of therapeutic intervention, we demonstrated the significant capability of the PRMT6 inhibitor, EPZ020411, to curtail breast cancer metastasis both in vivo and in vitro. In sum, our study unveils the pivotal biological role of PRMT6-mediated STAT3 R729me2a in breast cancer metastasis and underscores the prospective utility of PRMT6 inhibitors as effective therapeutic strategies against STAT3-driven metastatic breast cancer.

## Introduction

Breast cancer (BRCA) stands as the most prevalent malignancy, representing the leading cause of death among women [1, 2]. It surpassed lung cancer in 2020 to become the primary cause of cancer-related deaths in women [1]. In clinical practice, BRCA manifests in distinct subtypes defined by the expression status of estrogen receptor (ER), progesterone receptor (PR), and human epidermal receptor growth factor receptor 2 (HER2; also known as ERBB2) [3, 4]. The majority of BRCA patients exhibit ER-positive and/or PR-positive traits, showing favorable responsiveness to hormone therapy [5]. Meanwhile, HER2-positive patients constitute 15–20% of all BRCA cases, and for this subgroup, anti-HER2 antibodies (like Trastuzumab, Pertuzumab, and Neratinib) have demonstrated notable efficacy. Combining anti-HER2 treatments with chemotherapy has shown encouraging clinical outcomes [4–7]. On the other hand, there exist other cases of BRCA categorized as triple-negative breast cancer (TNBC), lacking expression of ER, PR, and HER2, encompassing around 10–20% of all BRCA instances [4, 8, 9]. TNBC earns distinction due to its heightened aggressiveness and bleak prognosis. Despite chemotherapy’s central role in TNBC treatment, relapse remains an unfortunate possibility for a subset of patients [5, 9, 10]. The challenges of TNBC-linked relapse and distant metastasis take center stage in the management of BRCA [11, 12]. This study delves into the intricate mechanisms of BRCA metastasis, aiming to furnish innovative insights that hold the potential to enhance BRCA therapies.

Histone Arginine N-Methyltransferase 6 (PRMT6) is a member of the arginine N-methyltransferase family. It facilitates the transfer of a methyl group from S-adenosyl-l-methionine to the side chain nitrogens of arginine residues within proteins [13]. Additionally, PRMT6 can catalyze the creation of omega-N mono-methylarginine (MMA) and, more commonly, the generation of asymmetrical dimethylarginine (aDMA) [14, 15]. Notably, PRMT6 is responsible for catalyzing the asymmetric di-methylation of histone H3R2 (H3R2me2a), which is mutually exclusive with H3K4me2 and H3K4me3, thus transcriptionally suppressing various gene expressions such as P53, THBS1, and HOXA2. PRMT6 also mediates H2AR3me and H4R3me [14–17]. Recently, an increasing number of studies have been dedicated to investigating PRMT6-mediated non-histone protein methylation. For instance, PRMT6-mediated DNA polymerase beta (POLB) R83 and R152 methylation enhanced DNA polymerase activation, augments DNA binding and processivity, and further regulates DNA repair [18]. PRMT6-methylated CRTC2 strengthened both CRTC2 and CREB binding on the promoter of gluconeogenic enzyme-encoding genes, thereby inducing fasting-induced transcriptional activation of the gluconeogenic program [19]. Methylation of SIRT7 at R388 suppressed SIRT7 histone deacetylase activity, consequently promoting mitochondria biogenesis [20]. Furthermore, PRMT6 methylation at RCC1 R214 has been shown to regulate the tumorigenicity and radiation response of glioblastoma stem cells [21]. Other proteins, such as GPS2, HIV-1, and TOP3B, could also undergo methylation by PRMT6, affecting various biological processes [22–24]. However, there is a dearth of research concerning the role of PRMT6 in BRCA and metastasis.

In this study, our objective was to uncover PRMT6’s biological role in BRCA progression. Our findings indicated that PRMT6 acted as an oncogene in BRCA. Its expression level positively correlated with the aggressiveness of BRCA cells. Moreover, high PRMT6 expression was associated with advanced N, M, and TNM stages. Furthermore, we discovered that PRMT6 regulated the IL-6/STAT3 signaling pathway by asymmetrically di-methylating STAT3 at arginine 729. This methylation was crucial for STAT3’s binding to JAK2 and its membrane localization. Notably, the PRMT6 inhibitor EPZ020411 effectively curbed BRCA lung metastasis. This discovery suggested a promising novel treatment strategy for BRCA.

## Material and methods

### Cell lines and agents

The HEK 293T, MCF-7, H1954, and MDA-MB-468 cell lines were procured from The Global Bioresource Center (ATCC). L15 medium supported the MDA-MB-468 cell culture. HEK 293T and MCF-7 cell lines were cultivated in Dulbecco’s modified Eagle’s (DMEM) medium (Thermo Fisher Scientific). H1954 cell lines were cultured in RPMI-1640 medium. All media were enriched with fetal bovine serum to attain a final concentration of 10% (v/v). MDA-MB-468 cell lines were cultured under 100% air conditions, whereas H1954, HEK 293T, and MCF-7 cell lines were maintained with a mixture of 5% CO_2_ and 95% air. Every cell line underwent authentication through STR profiling, and rigorous testing confirmed the absence of any mycoplasma contamination. Antibodies for PRMT6 (#14641; Cell Signaling Technology), β-actin (#3700; Cell Signaling Technology), STAT3 (#8768; Cell Signaling Technology), p-STAT3 (#4074; Cell Signaling Technology), E-cadherin (ab1416; Abcam), Vimentin (ab1416; Abcam), histone 3 (#4499; Cell Signaling Technology), GAPDH (sc-47724; Santa Cruz Biotechnology), HA-tag (sc-7392; Santa Cruz Biotechnology), flag (#14793; Cell Signaling Technology), and H4R3me2a Antibody (PA5-96124; Thermo Fisher Scientific) were procured from their respective sources. The antibody for STAT3 R729me2a was manufactured by ABclonal (China). The inhibitor EPZ020411 was obtained from MedChemExpress.

### Real-time PCR assays (RT-PCR)

Total RNAs from the cultured cells were isolated using TRIZOL (Thermo Fisher Scientific). cDNAs were synthesized using ABScript III RT Master Mix for qPCR with gDNA Remover (RK20429; Abclonal), following the manufacturer’s instructions. RT-PCR assays were conducted with 2X Universal SYBR Green Fast qPCR Mix (RK21203, Abclonal) on a QuantStudioTM 3 Real-Time PCR Instrument (Thermo Fisher Scientific). The primer sequences used were as follows: Twist (Forward: 5- GTCCGCAGTCTTACGAGGAG-3; Backwards: 5-GTCCGCAGTCTTACGAGGAG-3); E-cadherin (Forward: 5-CGAGAGCTACACGTTCACGG-3; Backwards: 5-GTCCGCAGTCTTACGAGGAG-3); N-cadherin (Forward: 5- TCAGGCGTCTGTAGAGGCTT-3; Backwards: 5-ATGCACATCCTTCGATAAGACTG-3); Vimentin (Forward: 5-GACGCCATCAACACCGAGTT-3; Backwards: 5-CTTTGTCGTTGGTTAGCTGGT-3); and GAPDH (Forward: 5-GGAGCGAGATCCCTCCAAAAT-3; Backwards: 5- GGCTGTTGTCATACTTCTCATGG-3).

### Transwell assays

A total of 2–6 × 10^5^ pre-treated cells were introduced into the upper chamber, with 300 µL of serum-free medium. Concurrently, the lower chamber received 400 µL of medium containing 10% (v/v) fetal bovine serum. Following an incubation period of 12–16 h, cells on the membrane were immobilized using 4% paraformaldehyde and subsequently stained with a 1% crystal violet staining solution. For inner membrane cells, a cotton swab was employed to gently remove them, after which they were visualized using an inverted microscope.

### Wound healing assays

Pre-treated cells were cultivated in six-well plates until they achieved a density of 95%. Subsequently, wound healing assays were conducted by gently creating wounds using a 10 μL pipette tip. The medium was then substituted with a serum-free medium, supplemented with Mitomycin (1 µg/mL) to impede cancer cell proliferation. Over the course of 0 to 24 h, the distance of wound closure was documented using an inverted microscope.

### Colony formation assays

Briefly, 300–600 pre-treated cells were introduced into a 24-well plate, and the medium was renewed every 3 days. Following a 14-day incubation period, colonies were immobilized using 4% paraformaldehyde and subsequently stained with a 1% crystal violet staining solution. The resulting colonies were then captured using an inverted microscope.

### Cell lines construction

For the construction of PRTM6 or STAT3 knockdown cell lines, sgRNAs were integrated into pSpCas9(BB)-2A-Puro (PX459) V2.0 plasmids. Subsequently, 1 µg of the plasmid was transfected into MCF-7 or MDA-MB-468 cells. Following a 48–72-h interval, puromycin (2 μg/mL) was applied to select cells until viability ceased in control cells. The remaining cells (~100 cells) were then seeded into a 96-well plate for monoclonal screening. Regarding the establishment of PRMT6 stably expressing cell lines, PRMT6 or PRMT6 KLA genes were incorporated into pLVX-vector plasmids. The lentivirus was generated using the PPAX, MD2-G, and pLVX three-package system. Subsequently, the lentivirus was introduced into MCF-7 and MDA-MB-468 cells. Following a 48-h period, puromycin (2 μg/mL) was used to screen cells until viability ceased in control cells. To confirm successful construction, Western blots and RT-PCR analyses were conducted.

### Western blots and immunoprecipitation assays

The collected cells were lysed with NP40 on ice for 15 min. Subsequently, centrifugation at 12,000 r.p.m. and 4 °C for 10 min was performed to isolate cell debris. Protein separation was accomplished via electrophoresis in precast sodium dodecyl sulfate–polyacrylamide minigels (Tris–HCl SDS–PAGE) and then transferred onto a PVDF membrane. Primary antibodies (dilution 1:1000) were incubated with the membranes overnight at 4 °C, followed by a subsequent incubation with HRP-conjugated secondary antibodies. Protein signaling was visualized using a chemiluminescent solution. In the context of immunoprecipitation assays, primary antibodies were initially incubated with Protein L Magnetic Beads (HY-K0205; MCE) at room temperature for 2 h. The lysate was subsequently exposed to the pre-treated magnetic beads and incubated overnight at 4 °C, followed by Western blot analysis.

### Nuclear and cytoplasmic protein extraction

Nuclear and cytoplasmic proteins were isolated using the Nuclear and Cytoplasmic Protein Extraction Kit (P0027, Beyotime). Subsequently, Western blot analysis was conducted on the separated nuclear and cytoplasmic protein fractions.

### Immunofluorescence assay

Pre-treated cells were initially seeded onto cell slides at a density of 30–50%. After IL-6 stimulation, the cells were fixed using 4% paraformaldehyde. Subsequently, 0.5% Triton X-100 was applied at room temperature for 10 min. Following this, 5% BSA was added to the cell slides at room temperature for 30 min. The slides were then incubated with the designated primary antibody overnight at 4 °C, followed by secondary antibody incubation. Images were captured using a confocal laser scanning microscope.

### Immunohistochemistry (IHC)

All clinic samples were obtained from BRCA patients who did not receive chemotherapy or radiotherapy before surgery at Wuhan Union Hospital. Two separate pathologists conducted an IHC analysis to evaluate the staining patterns of each sample. The IHC staining scores were evaluated using the IRS system. The percentage of positively stained cells was assigned scores as follows: 1 (<10%), 2 (10–50%), 3 (50–75%), and 4 (>75%). Staining intensity was scored on a scale of 0–3: 0 for no staining, 1 for light yellow, 2 for yellow-brown, and 3 for brown. The staining indices were determined by multiplying the percent score with the intensity score.

### Animal experiments

The animal study received ethical approval from the Institutional Animal Care and Use Committee at Tongji Medical College of Huazhong University of Science and Technology. The animal sample size was estimated according to a balance between having a sample size large enough to detect the desired effect while considering practical and ethical constraints. For the animal experiments, the three-blind principle is followed. Female mice aged 4–6 weeks were acquired from Beijing Huafukang Bioscience Company. The female mice were randomly assigned, and 1–2 × 10^6^ cells were suspended in 100 µL PBS and injected into the tail vein. After a span of 2 months, the mice were humanely euthanized. Subsequently, their lungs were extracted, weighed, and then fixed using 4% paraformaldehyde for subsequent analysis.

### Datasets

The GSE21653, GSE42568, and GSE2741 datasets were obtained from the TCGA database (https://www.ncbi.nlm.nih.gov/pubmed/). Subsequently, GSEA analysis was conducted using the GSEA software.

### Statistical analysis

The sample size of all experiments was chosen to strike a balance between having a sample size large enough to detect the desired effect while considering practical and ethical constraints. Statistical analysis was carried out using SPSS (v20.0) and GraphPad Prism (v9.0.0) software. The Student’s *t*-test was employed to compare differences between the two groups if the variance was similar between the groups. If else, the Mann–Whitney test was used. A *P* value < 0.05 was regarded as statistically significant.

## Results

### PRMT6 acted as an oncogene in BRCA

**Fig. 1:**
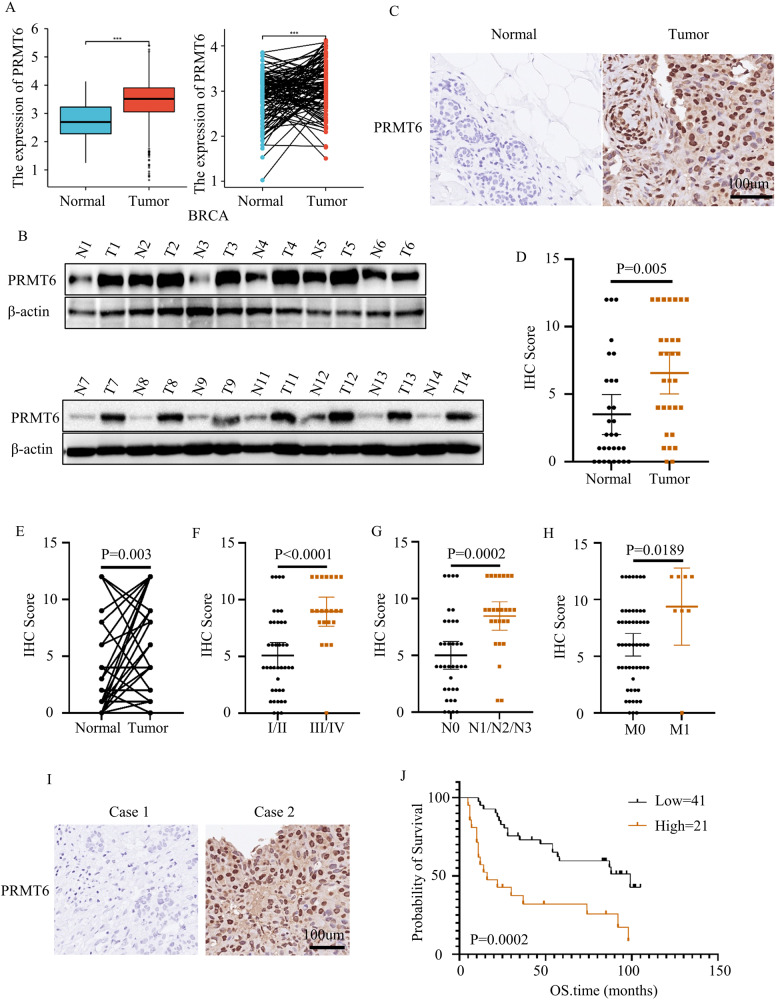
PRMT6 was upregulated in breast cancer. **A** Analysis of the expression level of PRMT6 in breast cancer from the TCGA database; left for unpaired cancer tissues, right for paired cancer tissues. **B** Analysis of the expression level of PRMT6 by immunoblotting assays. **C** Representative image for IHC assays in detecting the expression level of PRMT6 in breast cancer or normal adjacent tissues. **D** and **E** Statistical analysis for the expression level of PRMT6 in breast cancer tissues and adjacent normal tissues; *n* = 30, *P* < 0.05. **F**–**H** Statistical analysis for the expression level of PRMT6 in breast cancer patients grouped by N, M, or clinical stages; *n* = 62, *P* < 0.05. **I** Representative images of IHC assays in investigating the expression level of PRMT6 in breast cancer. **J** Kaplan–Meier Plot was drawn, patients were grouped by the expression level of PRMT6; *P* < 0.05. All immunoblotting assays were conducted three times, and consistent results were obtained.

In order to elucidate the underlying biological role of PRMT6 in BRCA, our initial investigation focused on the expression level of PRMT6 within BRCA, utilizing data from the TCGA database. The results notably revealed a significant upregulation of PRMT6 not only in BRCA tissues but also across various other cancer types (Fig. 1A and Supplementary Fig. 1A). To authenticate these findings, we conducted western blot assays on 14 pairs of BRCA tissues alongside their matched adjacent normal breast tissues, consistently yielding congruent outcomes (Fig. 1B). Subsequently, employing IHC, we probed the PRMT6 expression levels in 30 pairs of BRCA and adjacent normal tissues, achieving similar results (Fig. 1C–E). Furthermore, IHC was implemented to explore PRMT6’s expression across 62 instances of BRCA cases. Impressively, the results showcased heightened PRMT6 expression in patients with elevated N stage, M stage, or clinical stage (Fig. 1F–H). Supplementary Table 1 details the fundamental clinical data pertaining to the patients. Subsequently, the patient cohort was categorized based on their PRMT6 expression levels. Strikingly, Kaplan–Meier analysis demonstrated that individuals exhibiting elevated PRMT6 expression levels experienced shorter overall survival durations (Fig. 1I, J). Likewise, Kaplan–Meier Plotter database analysis reinforced these observations, illustrating an inverse correlation between PRMT6 expression levels and overall survival time across multiple cancer types (Supplementary Fig. 1B). In summation, these collective findings indicated that PRMT6 might exert a promotive influence on the progression of BRCA.

### PRMT6 positively regulated BRCA metastasis

**Fig. 2:**
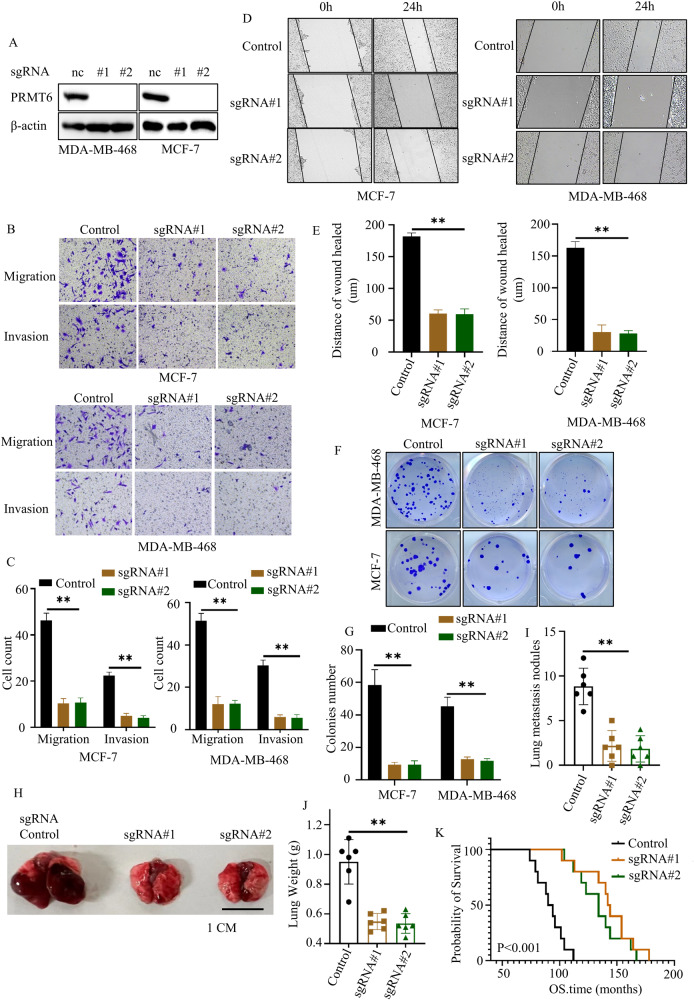
Loss of PRMT6 inhibited the migration, invasion, and colony formation ability of cancer cells. **A** Immunoblotting assay confirming the successful construction of identified cancer cells. **B** Representative image of transwell assays of cancer cells stably expressing sgRNA-Control, sgRNA-PRMT6#1, or sgRNA-PRMT6#2. **C** Statistical analysis for transwell assays in Fig. 2B; *n* = 3, *P* < 0.05. **D** Representative image of wound healing assays of cancer cells stably expressing sgRNA-Control, sgRNA-PRMT6#1, or sgRNA-PRMT6#2. **E** Statistical analysis for wound healing assays in **D**; *n* = 3, *P* < 0.05. **F** Representative image of colony formation assays of cancer cells stably expressing sgRNA-Control, sgRNA-PRMT6#1, or sgRNA-PRMT6#2. **G** Statistical analysis for colony formation assays in (**F**); *P* < 0.05. **H** Representative image of the mouse lung metastasis model. **I** Statistical analysis of lung metastasis nodules in **H**; *n* = 6, *P* < 0.05. **J** Statistical analysis of lung weight in **H**; *n* = 6, *P* < 0.05. **K** Kaplan–Meier plot was drawn in the three groups of mice lung metastasis assays; *n* = 10, *P* < 0.05. All immunoblotting assays were conducted three times, and consistent results were obtained.

As depicted in Fig. 1, a notable trend emerged wherein patients with higher N or M stages exhibited elevated PRMT6 expression levels. Given the significant challenge posed by BRCA-related distant metastasis in patient management [10], we set out to explore whether PRMT6 might play a role in the regulation of BRCA metastasis. To this end, we initiated our investigation by constructing stable MDA-MB-468 and MCF-7 cell lines expressing PRMT6 vectors (Supplementary Fig. 2A). Employing wound healing assays, we gauged the migration capabilities of these four cell variants. Encouragingly, the introduction of PRMT6 led to a discernible enhancement in cancer cell metastatic potential (Supplementary Fig. 2B, C). Concomitantly, we conducted Transwell assays to assess both migration and invasion abilities among the four cell types. The outcomes consistently indicated that heightened PRMT6 levels correlated with notable increases in both migration and invasion capacities (Supplementary Fig. 2D, E). Recognizing the pivotal role of ectopic colonization as the inaugural step in the distant metastasis of cancer cells, we performed colony formation assays to quantify the colony-forming aptitude of these four cancer cell types. Significantly, cells stably expressing PRMT6 demonstrated an augmented ability to generate colonies (Supplementary Fig. 2F, G). To validate the aforementioned findings, we employed Crispr-Cas9 gene editing to knock out PRMT6 in MCF-7 and MDA-MB-468 cells (Fig. 2A). Encouragingly, the outcomes of wound healing, Transwell, and colony formation assays were congruent, establishing that PRMT6 loss effectively inhibited cancer cell migration, invasion, and colony formation (Fig. 2B–G). Besides, Cells harboring sgRNA-PRMT6#1 and sgRNA-PRMT6#2 exhibited an epithelial morphology characterized by preserved cell–cell adhesions and a cobblestone-like appearance (Supplementary Fig. 2H). Subsequently, a mouse model of lung metastasis was created using MDA-MB-468 cells engineered to express sgRNA-control, sgRNA-PRMT6#1, and sgRNA-PRMT6#2. To achieve this, cancer cells were injected into the tail veins of 6-week-old female nude mice. After 2 months, six mice from each group were sacrificed. Their lungs were harvested, weighed, and the survival duration of the remaining mice was monitored until the study’s conclusion. The results compellingly demonstrated that PRMT6 knock-out significantly curtailed cancer cell metastasis, leading to a prolongation in the overall survival time of mice (Fig. 2H–K). Collectively, these findings substantiated PRMT6 as a contributory factor to BRCA cell metastasis.

### PRMT6 positively regulated IL-6/STAT3 signaling pathway

**Fig. 3:**
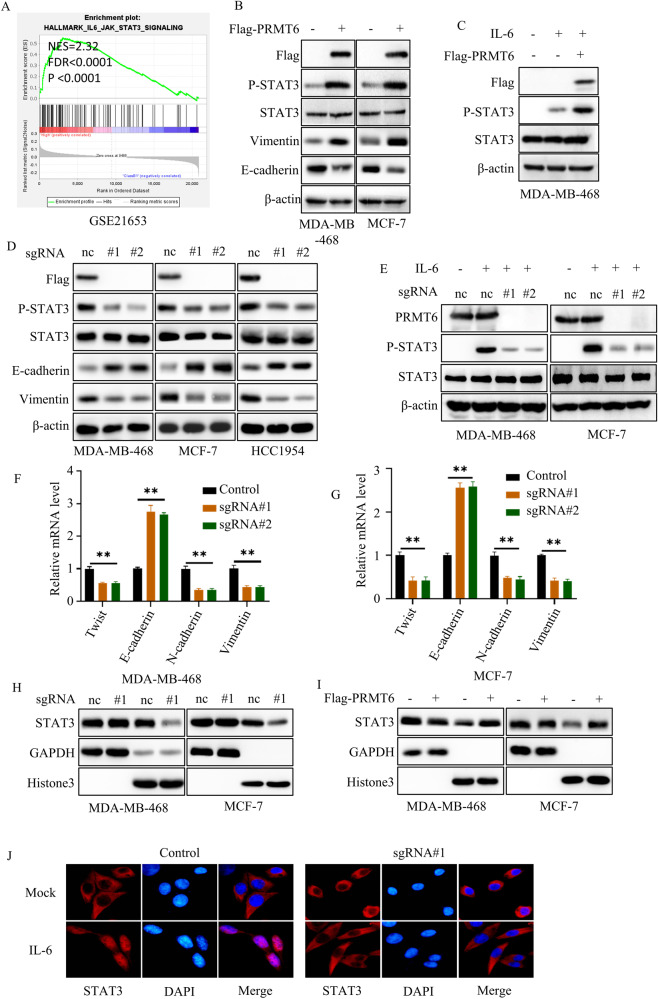
PRMT6 positively regulated the IL-6/STAT3 signaling pathway. **A** GSEA analysis for GSE21653, patients grouped by the expression level of PRMT6. **B** Immunoblotting assays to detect the expression level of identified protein in cancer cells stably expressing Vector or PRMT6. **C** Immunoblotting assays to detect the expression level of STAT3 phosphorylation in cancer cells stably expressing Vector or PRMT6 with or without IL-6 (10 ng/mL) stimulation. **D** Immunoblotting assays to detect the expression level of identified protein in cancer cells stably expressing sgRNA-Control, sgRNA-PRMT6#1, or sgRNA-PRMT6#2. **E** Immunoblotting assays to detect the expression level of STAT3 phosphorylation in cancer cells stably expressing sgRNA-Control, sgRNA-PRMT6#1, or sgRNA-PRMT6#2 with or without IL-6 (10 ng/ml) stimulation. **F** and **G** RT-PCR assays to investigate the mRNA expression level of epithelial-mesenchymal transition markers in cancer cells stably expressing sgRNA-Control, sgRNA-PRMT6#1, or sgRNA-PRMT6#2; *n* = 6, *P* < 0.05. **H** Isolating cytoplasm and nucleus, immunoblotting assays to detect the expression level of STAT3 in cancer cells stably expressing sgRNA-Control or sgRNA-PRMT6#1. **I** After isolating the cytoplasm and nucleus, immunoblotting assays was performed to detect the expression level of STAT3 in cancer cells stably expressing Vector or PRMT6. **J** Immunofluorescence assays to assess the STAT3 cellular localization in cancer cells stably expressing sgRNA-Control or sgRNA-PRMT6#1 with or without IL-6 (10 ng/mL) stimulation. All immunoblotting assays were conducted three times, and the results were consistent.

In this context, we delved into the intrinsic mechanism underlying PRMT-mediated tumor metastasis. Leveraging GSEA analysis on the GSE21653, GSE42568, and GSE2741 datasets, we unearthed a compelling correlation: patients exhibiting elevated PRMT6 expression levels demonstrated heightened activation of the IL-6/STAT3 signaling pathway (Fig. 3A and Supplementary Fig. 3A). Subsequently, immunoblotting analysis revealed that heightened PRMT6 expression resulted in amplified Y705 phosphorylation levels of STAT3 (an indicator of IL-6/STAT3 pathway activation), accompanied by increased Vimentin expression levels and suppressed E-cadherin expression levels (markers indicative of epithelial–mesenchymal transition) (Fig. 3B, C). Supporting this, RT-PCR assays affirmed that increased PRMT6 levels correlated with elevated mRNA expression of twist, N-cadherin, and Vimentin, along with downregulated mRNA expression of E-cadherin (Supplementary Fig. 3B). Impressively, consistent findings emerged across sgRNA-Control, sgRNA-PRMT6#1, and sgRNA-PRMT6#2 cancer cells (Fig. 3D–G).

Earlier research emphasized the essential role of Y705 phosphorylation in mediating STAT3’s nuclear localization. To validate this, we isolated cytoplasmic and nuclear fractions from sgRNA-Control and sgRNA-PRMT6#1 cancer cells. Subsequent immunoblotting assays were conducted to gauge the STAT3 expression levels in these cell types. Significantly, sgRNA-PRMT6#1 cancer cells displayed diminished nuclear STAT3 expression compared to sgRNA-Control cells (Fig. 3H). Additionally, cells overexpressing PRMT6 exhibited higher nuclear STAT3 expression compared to vector control cells (Fig. 3I). Immunofluorescence assays further supported these observations, showcasing the translocation of STAT3 into the nucleus of sgRNA-Control cells under IL-6 stimulation, while this event was not replicated in sgRNA-PRMT6 cells (Fig. 3J). In sum, these comprehensive results collectively substantiated PRMT6’s capacity to positively modulate the IL-6/STAT3 signaling pathway.

### STAT3 was a key factor for PRMT6-mediated tumor metastasis

**Fig. 4:**
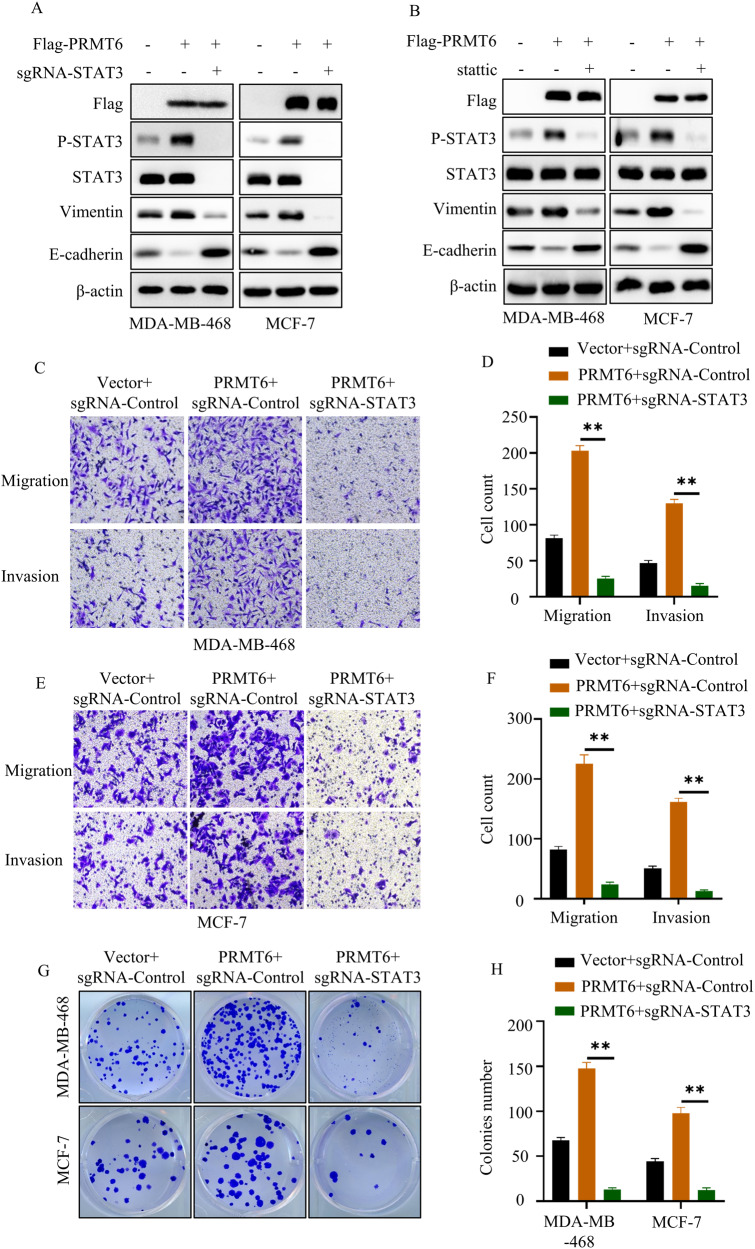
PRMT6-mediated cancer cell metastasis relied on STAT3 signaling. **A** Immunoblotting assay to detect the expression level of identified protein in cancer cells stably expressing Vector + sgRNA-Control, PRMT6 + sgRNA-Control, and PRMT6 + sgRNA-STAT3. **B** Immunoblotting assays to detect the expression level of identified protein in cancer cells stably expressing Vector or PRMT6 with or without stattic (20 µM). **C**, **E** Representative image of trans-well assays of cancer cells stably expressing Vector + sgRNA-Control, PRMT6 + sgRNA-Control, and PRMT6 + sgRNA-STAT3. **D**, **F** Statistical analysis for transwell assays in **C** or **E**; *n* = 3, *P* < 0.05. **G** Representative image of colony formation assays of cancer cells stably expressing Vector + sgRNA-Control, PRMT6 + sgRNA-Control, and PRMT6 + sgRNA-STAT3. **H** Statistical analysis for colony formation assays in **G**; *n* = 3, *P* < 0.05. All immunoblotting assays were conducted three times, and the results were consistent.

Having established PRMT6’s capacity to regulate the IL-6/STAT3 signaling pathway—an essential player in tumor metastasis—it was imperative to delve deeper into whether PRMT6-mediated tumor metastasis hinged on the IL-6/STAT3 signaling pathway. To ascertain this, we initially engineered BRCA cells to stably express Vector + sgRNA-Control, PRMT6 + sgRNA-Control, and PRMT6 + sgRNA-STAT3. Through immunoblotting assays, we observed that the increased STAT3 Y705 phosphorylation levels, heightened vimentin levels, and diminished E-cadherin expression resulting from PRMT6 overexpression could be effectively countered by the depletion of STAT3 (Fig. 4A).

Subsequently, we employed stattic—an inhibitor targeting the Y705 phosphorylation of STAT3. Immunoassays unveiled that the PRMT6-induced elevation in STAT3 Y705 phosphorylation levels, augmented vimentin levels, and decreased E-cadherin expression could be reversed through stattic administration (Fig. 4B). Moreover, Transwell assays were employed to gauge the migration and invasion potential of cancer cells stably expressing Vector + sgRNA-Control, PRMT6 + sgRNA-Control, and PRMT6 + sgRNA-STAT3. Notably, the heightened migration and invasion abilities resulting from PRMT6 upregulation were effectively countered by STAT3 silencing (Fig. 4C–F). Notably, consistent findings were replicated in cancer cells treated with Vector + DMSO, PRMT6 + DMSO, and PRMT6 + stattic (Supplementary Fig. 4A–D). Colony formation assays were also conducted to quantify the colony-forming potential of cancer cells stably expressing Vector + sgRNA-Control, PRMT6 + sgRNA-Control, and PRMT6 + sgRNA-STAT3. Impressively, the upregulation of colony-forming capabilities induced by PRMT6 could be negated by STAT3 inhibition (Fig. 4G, H). In summation, these comprehensive results underscored that PRMT6-mediated tumor cell metastasis indeed hinges on STAT3 activation.

### PRMT6 asymmetrically di-methylated STAT3 at arginine 729

**Fig. 5:**
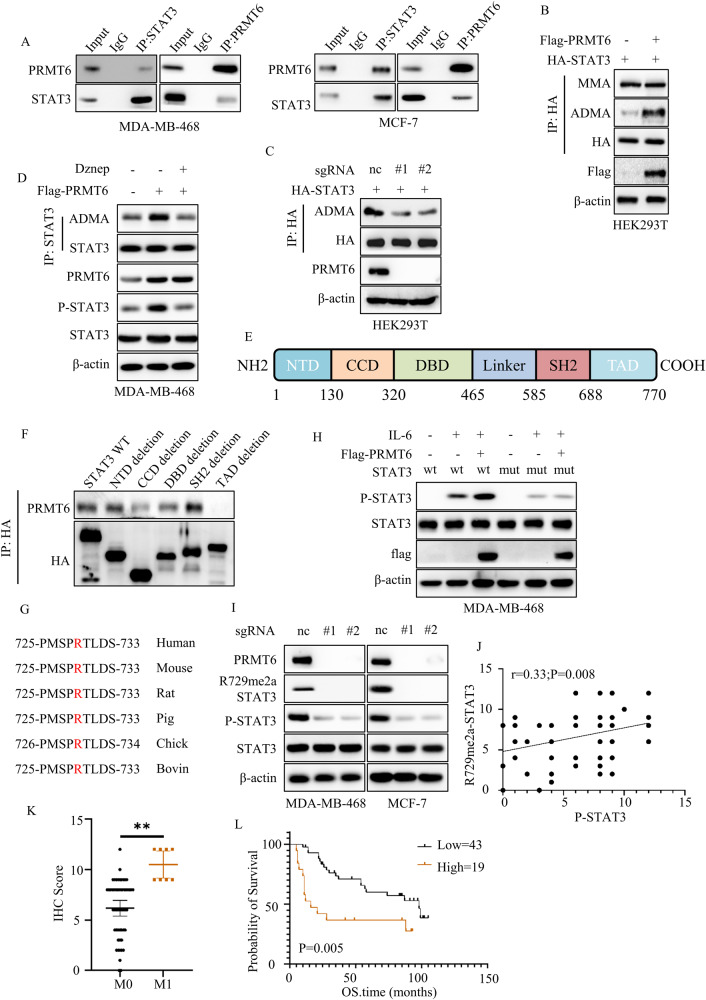
STAT3 R729 was asymmetrically di-methylated by PRMT6. **A** Co-immunoprecipitation assays to investigate the binding of PRMT6 and STAT3 in MCF-7 and MDA-MB-468 cancer cells. **B** Immunoblotting assays to detect the MMA or ADMA expression level of STAT3 in cancer cells stably expressing Vector or PRMT6. **C** Immunoblotting assays to detect the ADMA expression level of STAT3 in cancer cells stably expressing sgRNA-Control, sgRNA-PRMT6#1, or sgRNA-PRMT6#2. **D** Immunoblotting assays to detect the ADMA expression level of STAT3 in cancer cells stably expressing Vector or PRMT6 with or without Dznep treatment. **E** The model of STAT3 structure domains. **F** Transfecting HA-tagged segmented plasmids of STAT3 into HEK293T cells, co-immunoprecipitation assays to investigate the binding of PRMT6 and STAT3. **G** The sequence from 725 to 733 amino acids of STAT3 in various species. **H** Immunoblotting assays to investigate the STAT3 phosphorylation level of cancer cells stably expressing STAT3 WT+ vector, STAT3 WT + PRMT6, STAT3 R729K+ vector, or STAT3 R729K + PRMT6 with or without IL-6 (10 ng/mL) stimulation. **I** Immunoblotting assays to investigate the expression level of identified protein in cancer cells stably expressing sgRNA-Control, sgRNA-PRMT6#1, or sgRNA-PRMT6#2. **J** Scatter plot showing the expression level of STAT3 phosphorylation and STAT3 R729 methylation; *n* = 62, *P* < 0.05. **K** Statistical analysis of the expression level of STAT3 R729 methylation in breast cancer patients grouped by M stage; *n* = 62, *P* < 0.05. **L** The patients were grouped by the expression level of STAT3 R729 methylation, and the Kaplan–Meier Plot was drawn; *P* < 0.05. All immunoblotting assays were conducted three times, and the results were the same.

We initiated our inquiry by analyzing the STRING database to unravel the mechanics of PRMT6-mediated activation of the IL-6/STAT3 signaling pathway. Remarkably, our findings highlighted an interaction between PRMT6 and STAT3 (Supplementary Fig. 5a). Subsequently, co-immunoprecipitation assays were undertaken to ascertain whether PRMT6 could physically bind to STAT3, and the results disclosed a substantive interaction between PRMT6 and STAT3 in MCF-7 and MDA-MB-468 cancer cells (Fig. 5a). In consideration of previous literature, where PRMT6 was identified as an arginine N-methyltransferase capable of catalyzing both mono-methylarginine (MMA) and asymmetrical dimethylarginine (ADMA) formation—arginine methylation influencing protein activation, stabilization, and subcellular localization [13, 25, 26]—we conjectured that PRMT6 could catalyze arginine methylation on STAT3, consequently influencing its phosphorylation. To substantiate this hypothesis, we initially measured the levels of MMA and ADMA in STAT3 within cells expressing Vector, PRMT6 WT, or PRMT6 KLA mutant (an enzyme-deficient variant) [13]. Interestingly, we found that heightened PRMT6 WT expression, but not PRMT6 KLA, elevated the ADMA levels in STAT3, while the MMA levels remained unaffected (Fig. 5B and Supplementary Fig. 5B). Moreover, loss of PRMT6 significantly dampened STAT3’s ADMA levels (Fig. 5C). Concurrently, employing EPZ020411 (EPZ) [21], an inhibitor of PRMT6, substantially curtailed STAT3’s ADMA levels (Supplementary Fig. 5C).

Subsequently, we probed the correlation between PRMT6-mediated STAT3 methylation and STAT3 Y705 phosphorylation. Employing a global methyltransferase inhibitor, Dznep, we suppressed STAT3 methylation and observed through immunoblotting that PRMT6-induced upregulation in ADMA and phosphorylation of STAT3 could be abrogated by Dznep (Fig. 5D). Collectively, these findings implied that PRMT6 had the capacity to modulate STAT3 phosphorylation through arginine methylation.

Turning our focus to identifying the specific arginine residue within STAT3 targeted by PRMT6, we designed five segmented STAT3 plasmids based on their structural domains, each lacking one domain (Fig. 5E, F). Co-immunoprecipitation assays were conducted to elucidate which domain of STAT3 interacted with PRMT6. The outcomes revealed that the TAD domain’s deletion abrogated PRMT6–STAT3 interaction (Fig. 5F), signifying that PRMT6 likely targeted STAT3 for methylation at its TAD domain. Further scrutiny of the TAD domain’s amino acid sequence uncovered a solitary arginine (R729) amenable to methylation by PRMT6. This residue, R729, was found conserved across several species (Fig. 5G). To validate this, we created a HA-tagged STAT3 R729K mutant plasmid and co-transfected it with Flag-tagged PRMT6 or STAT3 WT plasmids into HEK293T cells. Subsequent immunoprecipitation and immunoblotting assays verified that PRMT6 overexpression increased ADMA levels in STAT3 WT but not STAT3 R729K mutant, where the latter exhibited notably lower ADMA levels (Supplementary Fig. 5D).

Subsequently, using CRISPR-Cas9 gene editing technology and lentivirus, we established MDA-MB-468 cells with stably expressing STAT3 WT or STAT3 R729K mutant (Supplementary Fig. 5E, F). Lentiviral constructs were also employed to generate MDA-MB-468 cells stably expressing STAT3 WT+ vector, STAT3 WT + PRMT6, STAT3 R729K+ vector, and STAT3 R729K + PRMT6. Immunoblotting assays were executed to assess the Y705 phosphorylation levels of STAT3 in these cell types under IL-6 stimulation. The results disclosed that PRMT6 overexpression amplified IL-6-induced Y705 phosphorylation in STAT3 WT, yet this effect was nullified in STAT3 R729K mutant, despite the latter having lower basal Y705 phosphorylation levels than STAT3 WT in normal culture conditions (Fig. 5H and Supplementary Fig. 5F). Moreover, specific asymmetric dimethylarginine antibody (R729me2a) was employed to measure STAT3’s R729me2a levels in cancer cells expressing Vector, PRMT6 WT, or PRMT6 KLA mutant. These assays revealed that PRMT6 WT, but not PRMT6 KLA, markedly upregulated STAT3’s R729me2a levels (Supplementary Fig. 5G). The loss of PRMT6 or administration of EZP exhibited similar inhibitory effects on STAT3’s R729me2a levels in vitro (Fig. 5I and Supplementary Fig. 5H). Importantly, in vivo, PRMT6 knock-out consistently hindered STAT3 phosphorylation and methylation (Supplementary Fig. 2I). To further corroborate the correlation between PRMT6 and STAT3, we conducted IHC assays on 62 BRCA tissue cases to gauge the expression levels of PRMT6, Y705 phosphorylated STAT3, and R729me2a. Notably, we observed positive correlations between PRMT6 expression and Y705 phosphorylation or R729me2a levels of STAT3 (Supplementary Fig. 5I, J). Moreover, R729me2a levels displayed a significant relationship with T705 phosphorylation (Fig. 5J). Importantly, we noted that patients with elevated N, M, or clinical stages exhibited higher R729me2a expression levels (Fig. 5K and Supplementary Fig. 5K). Furthermore, analysis of the Kaplan–Meier plot highlighted that patients with higher R729me2a expression levels experienced poorer overall survival (Fig. 5L). In conclusion, our study elucidated that PRMT6 positively modulated STAT3’s Y705 phosphorylation through asymmetric dimethylation of STAT3’s R729 residue. Moreover, we proposed that STAT3 R729 asymmetrical demethylation could serve as a predictive factor for the overall survival of BRCA patients.

### STAT3 R729 methylation was essential for its membrane localization

**Fig. 6:**
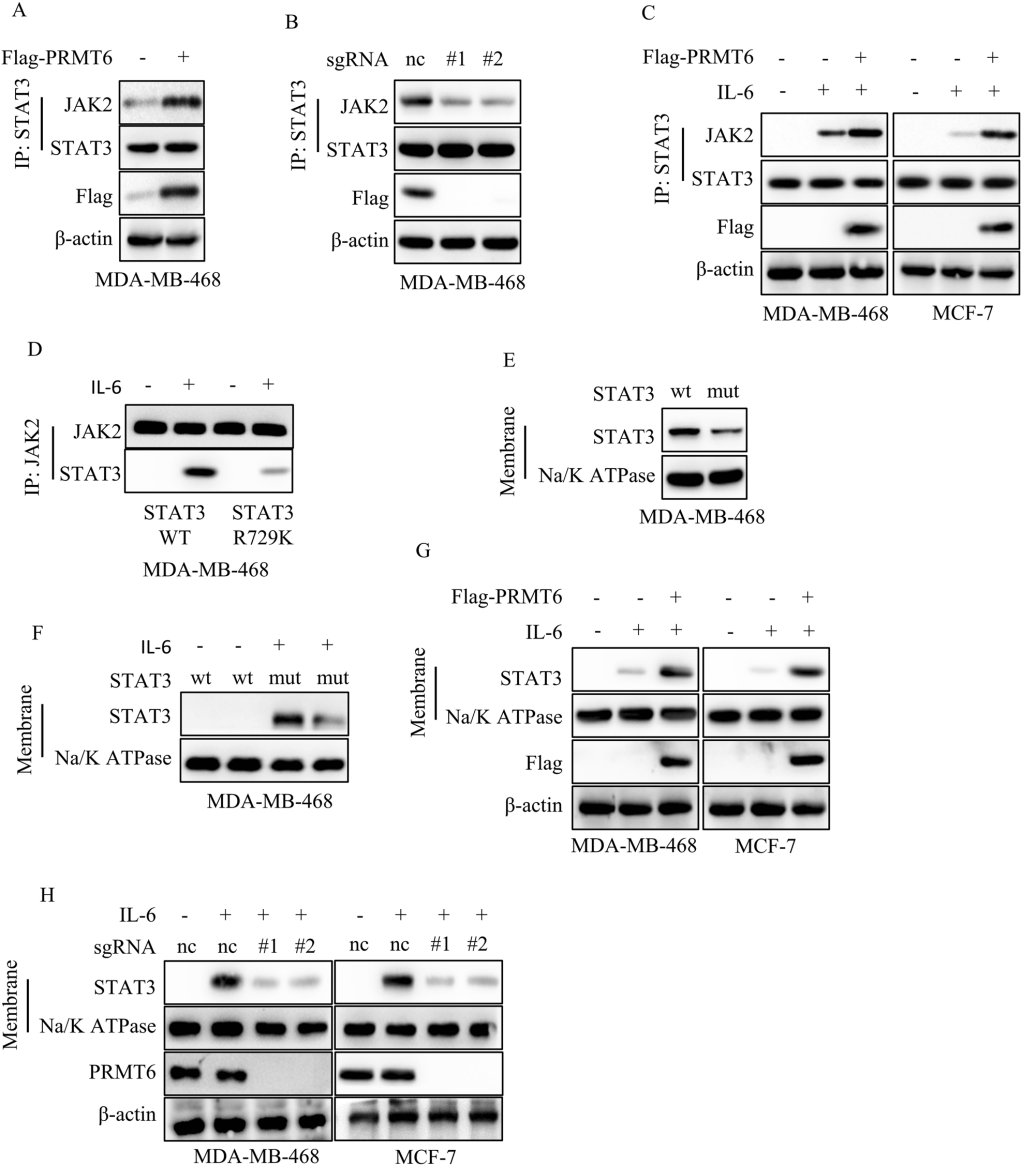
STAT3 R729 methylation was necessary for its membrane localization. **A** Immunoblotting and immunoprecipitation assays to detect the binding of STAT3 and JAK2 in cancer cells stably expressing Vector or PRMT6. **B** Immunoblotting and immunoprecipitation assays to detect the binding of STAT3 and JAK2 in cancer cells stably expressing sgRNA-Control, sgRNA-PRMT6#1, or sgRNA-PRMT6#2. **C** Immunoblotting and immunoprecipitation assays to detect the binding of STAT3 and JAK2 in cancer cells stably expressing Vector or PRMT6 with or without IL-6 (10 ng/ml) stimulation. **D** Immunoblotting and immunoprecipitation assays to detect the binding of JAK2 and STAT3 WT or STAT3 R729K mutant. **E** Isolating the cellular membrane, immunoblotting assays to detect the expression level of STAT3 in cancer cells stably expressing STAT3 WT or STAT3 R729K mutant. **F** Isolating the cellular membrane, immunoblotting assays to detect the expression level of STAT3 in cancer cells stably expressing STAT3 WT or STAT3 R729K mutant with or without IL-6 (10 ng/mL) stimulation. **G** Isolating the cellular membrane, immunoblotting assays to detect the expression level of STAT3 in cancer cells stably expressing Vector or PRMT6 with or without IL-6 (10 ng/mL) stimulation. **H** Isolating the cellular membrane, immunoblotting assays to detect the expression level of STAT3 in cancer cells stably expressing sgRNA-Control, sgRNA-PRMT6#1, or sgRNA-PRMT6#2 with or without IL-6 (10 ng/mL) stimulation. All immunoblotting assays were conducted three times, and the results were the same.

In our pursuit of understanding the intricate mechanisms underlying the impact of STAT3 R729 methylation on STAT3 Y705 phosphorylation, we delved into the interplay between STAT3 and JAK2, known to be essential for the activation of STAT3. As per prior reports, STAT3 serves as a substrate for JAK2, which initiates the phosphorylation cascade at the cellular membrane [27]. Following this phosphorylation, STAT3 forms homodimers or heterodimers, which subsequently translocate into the nucleus to regulate target gene promoters [28]. Drawing on this knowledge, we formulated the hypothesis that PRMT6-mediated STAT3 R729 methylation could potentially influence the binding of STAT3 and JAK2. To investigate this, we probed the interaction between STAT3 and JAK2 in cancer cells expressing either Vector or PRMT6. Remarkably, we discovered that PRMT6 overexpression heightened the interaction between STAT3 and JAK2 (Fig. 6A). This observation was consistent with our finding that the loss of PRMT6 interfered significantly with the binding of STAT3 and JAK2 (Fig. 6B). Moreover, our experiments unveiled that IL-6 stimulation enhanced the interaction between JAK2 and STAT3, which was further potentiated by PRMT6 overexpression (Fig. 6C).

Further scrutinizing the interaction between JAK2 and either STAT3 WT or STAT3 R729K mutant, we found that IL-6 significantly amplified the interaction between STAT3 WT and JAK2, while the presence of STAT3 R729K mutant notably disrupted this interaction, diminishing its IL-6-induced enhancement (Fig. 6D). Expanding our investigation to STAT3’s membrane localization, we isolated cellular membranes to examine how PRMT6-mediated STAT3 methylation regulated this process. Our results indicated that the loss of STAT3 R729 methylation impeded both basal and IL-6-induced STAT3 membrane recruitment (Fig. 6E, F). Furthermore, PRMT6 overexpression enhanced IL-6-mediated STAT3 membrane recruitment, while PRMT6 loss hindered this process (Fig. 6G, H). Together, our findings established that PRMT6-catalyzed STAT3 R729 methylation played a critical role in mediating the interaction between STAT3 and JAK2.

### PRMT6-mediated tumor metastasis relied on STAT3 R729 methylation

**Fig. 7:**
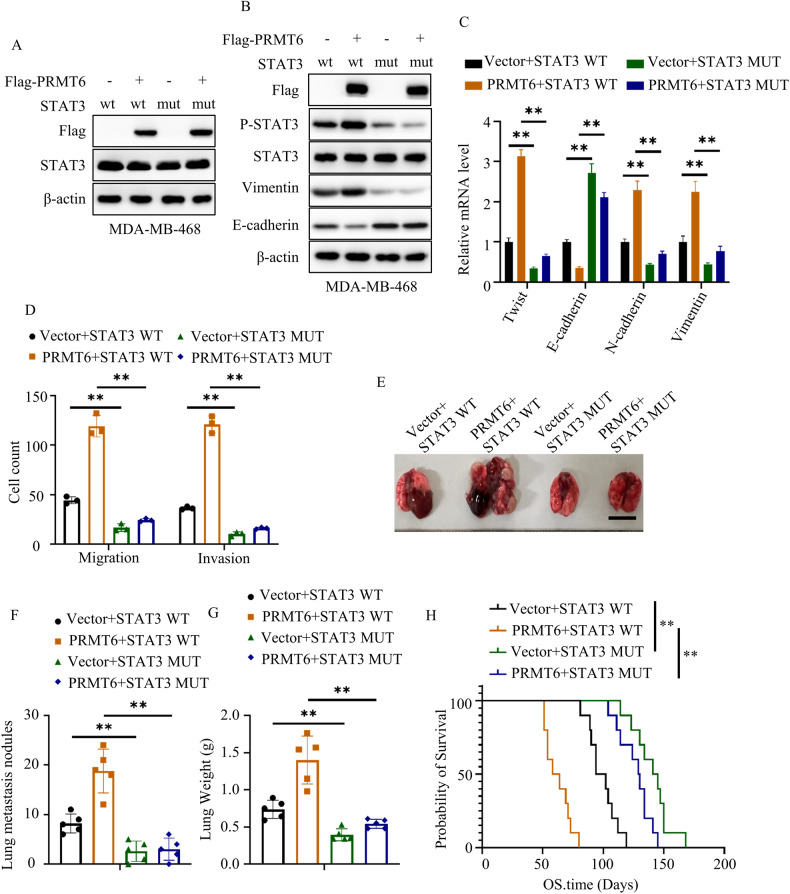
PRMT6-mediated tumor metastasis relied on STAT3 R729 methylation. **A** Immunoblotting assay confirming the successful construction of cancer cells stably expressing STAT3 WT+ vector, STAT3 WT + PRMT6, STAT3 R729K+ vector, and STAT3 R729K + PRMT6. **B** Immunoblotting assays to detect the expression level of targeting proteins in cancer cells stably expressing STAT3 WT+ vector, STAT3 WT + PRMT6, STAT3 R729K+ vector, and STAT3 R729K + PRMT6. **C** RT-PCR assays to detect the mRNA expression level of markers of epithelial–mesenchymal transition of cancer cell stably expressing STAT3 WT+ vector, STAT3 WT + PRMT6, STAT3 R729K+ vector, and STAT3 R729K + PRMT6; *n* = 3, *P* < 0.05. **D** Statistical analysis for trans-well assays in Supplementary Fig. 6A; *n* = 3, *P* < 0.05. **E** Representative image of mouse lung metastasis experiments. **F** Statistical analysis of lung nodules in **D**; *n* = 5, *P* < 0.05. **G** Statistical analysis of the weight of the mice lungs in **D**; *n* = 5, *P* < 0.05. **H** Kaplan–Meier Plot was drawn in four groups of mouse lung metastasis assays; *n* = 10, *P* < 0.05. All immunoblotting assays were conducted three times, and the results were the same.

To delve deeper into the intricate interplay between PRMT6 and STAT3 R729 methylation in the context of tumor metastasis, we engineered MDA-MB-468 cancer cells to stably express various combinations: STAT3 WT+ vector, STAT3 WT + PRMT6, STAT3 R729K+ vector, and STAT3 R729K + PRMT6 (Fig. 7A). Our subsequent investigations focused on the epithelial–mesenchymal transition (EMT) markers through immunoblotting and RT-PCR assays. Our findings highlighted that PRMT6 overexpression significantly elevated the expression of Vimentin, twist, and N-cadherin, while concurrently downregulating E-cadherin expression in cancer cells expressing STAT3 WT, but not in those expressing STAT3 R729K mutant (Fig. 7B, C). Transwell assays further bolstered our observations, showing that PRMT6 enhancement distinctly amplified the migration and invasion capabilities of cancer cells with STAT3 WT, yet failed to manifest this effect in cells with STAT3 R729K mutant (Fig. 7D and Supplementary Fig. 6A). To bridge our insights from in vitro assays to the in vivo context, we injected these four groups of cancer cells into the tail veins of 6-week-old female mice. Our subsequent xenograft study underscored that PRMT6 overexpression led to a significant increase in lung metastasis nodes and weight while shortening the overall survival time of mice harboring STAT3 WT cancer cells. Interestingly, this effect was notably dampened in mice with STAT3 R729K mutant cancer cells (Fig. 7E–H). In summation, our comprehensive findings provided robust confirmation that STAT3 R729 methylation constituted an indispensable factor in PRMT6-mediated tumor metastasis.

### PRMT6 was a potential target of STAT3-driven tumor metastasis

**Fig. 8:**
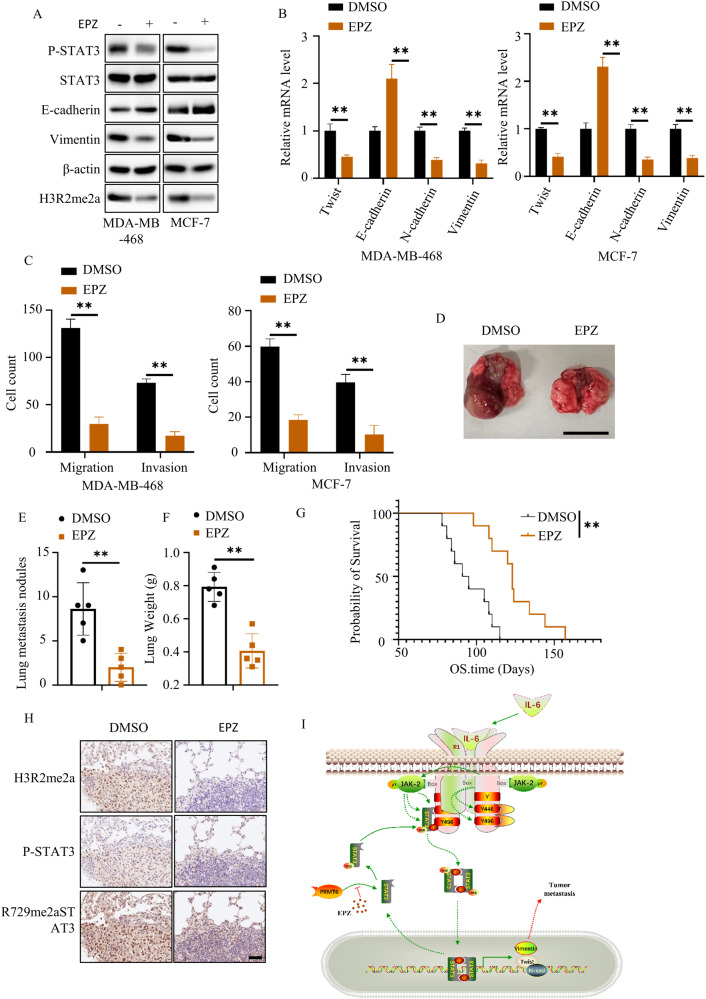
PRMT6 inhibitors were a potential therapeutic strategy for breast cancer. **A** Immunoblotting assay to detect the targeting proteins expression level in cancer cells with or without EPZ (20 µM) application. **B** RT-PCR assays to detect the mRNA expression level of marks of epithelial–mesenchymal transition of cancer cells with or without EPZ (20 µM); *n* = 3, *P* < 0.05. **C** Statistical analysis of transwell assays in Supplementary Fig. 6B, C; *n* = 3, *P* < 0.05. **D** Representative image of mouse lung metastasis experiments. **E** Statistical analysis of lung nodules in **D**; *n* = 5, *P* < 0.05. **F** Statistical analysis of the weight of the mice lungs in **D**; *n* = 5, *P* < 0.05. **G** Kaplan–Meier Plot was drawn for the two groups of mouse lung metastasis assays; *n* = 10, *P* < 0.05. **H** IHC assays to investigate the methylation and phosphorylation level of STAT3 in vivo xenograft. **I** The working model of PRMT6-mediated STTA3 signaling activation. All immunoblotting assays were conducted three times, and the results were the same.

Acknowledging the pivotal role played by the IL-6/STAT3 signaling pathway in tumor progression, efforts to hinder its hyperactivity have involved the exploration of therapeutic strategies such as IL-6 receptor mono-antibodies or STAT3 inhibitors. However, the clinical application of these drugs has been constrained by their associated side effects in cancer patients [28–30]. In light of this challenge, our investigation into the interaction of PRMT6 with JAK2 to activate the STAT3 pathway (Fig. 6) led us to consider PRMT6 inhibitors as a potentially effective approach for cancer treatment. By targeting PRMT6, these inhibitors could simultaneously disrupt both the H3R2-mediated epigenetic and IL-6/STAT3 oncogenic pathways. This concept was underscored by our in vivo results, wherein the deletion of PRMT6 significantly curtailed tumor metastasis (Fig. 2H–K). EPZ, a selective PRMT6 inhibitor, emerged as a viable candidate for intervention. Our observations indicated that EPZ could effectively suppress both STAT3 phosphorylation and H3R2 methylation (Fig. 8A), subsequently countering the epithelial-mesenchymal transition in cancer cells (Fig. 8A, B). In vitro assessments further substantiated these findings, demonstrating EPZ’s ability to hinder the migratory and invasive potential of cancer cells (Fig. 8C and Supplementary Fig. 6B, C). Moreover, the implementation of EPZ in a mouse model of lung metastasis, employing MDA-MB-468 cancer cells, revealed promising outcomes. The administration of EPZ at a dose of 10 mg/kg, through daily subcutaneous administration, led to a substantial reduction in lung metastasis nodules and an extension in overall survival among the mice subjected to MDA-MB-468 cancer cell xenografts (Fig. 8D–G). Notably, EPZ administration also yielded an inhibition of STAT3 methylation and phosphorylation in the in vivo context (Fig. 8H). Collectively, our findings illuminate the potential benefits of targeting PRMT6 as a therapeutic strategy to counteract both the STAT3 signaling pathway and the H3R2-mediated epigenetic mechanisms, offering new avenues for enhancing cancer treatment outcomes.

## Discussion

Tumor metastasis stands as a predominant cause of cancer-associated mortality [9]. PRMT6, characterized as a type I protein arginine methyltransferase (PRMT), exhibits the ability to perform asymmetric dimethylation of arginine residues on protein substrates, thereby orchestrating the epigenetic regulation of various gene expressions [13, 14]. In the context of breast cancer (BRCA), PRMT6’s engagement with the p21 promoter has been reported, resulting in the inhibition of p21 expression and the modulation of cellular senescence [31]. Additionally, the concerted actions of PRMT1, CARM1, and PRMT6 were implicated in driving BRCA progression through the regulation of H3R2 methylation [14, 32]. However, the precise role of PRMT6 in the context of BRCA metastasis remained to be elucidated. This present study, having been undertaken, aimed to address these gaps in understanding. Our investigation validated that PRMT6 indeed operates as an oncogene in the context of BRCA. We established that PRMT6 was markedly upregulated in BRCA tissues when contrasted with normal breast tissues. Importantly, patients with elevated PRMT6 expression exhibited higher N and M stages, alongside reduced overall survival rates (Fig. 1). In-depth exploration encompassing both in vitro and in vivo experiments demonstrated, for the first time, that the loss of PRMT6 led to a substantial inhibition of BRCA metastasis (Fig. 2). These collective findings coalesce to suggest the potential therapeutic viability of targeting PRMT6 for the management of BRCA.

The accumulated body of evidence underscores the pivotal role of the IL-6/STAT3 signaling pathway in steering tumor metastasis and invasion. This is predominantly achieved by orchestrating the expression of a spectrum of epithelial–mesenchymal transition (EMT)-related proteins, including but not limited to ZEB1, Snail, TWIST, Vimentin, and N-cadherin, while concurrently repressing the expression of counteracting EMT-related molecules such as E-cadherin [29, 30]. Within the framework of this signaling axis, STAT3 assumes a central role, as it serves as the linchpin of the IL-6/STAT3 cascade. Elucidating the precise regulatory mechanisms governing STAT3’s behavior thus becomes paramount. Our investigation added clarity to this narrative by revealing that PRMT6 exerted a positive influence on the activation of the IL-6/STAT3 pathway. This was achieved through its impact on STAT3 phosphorylation and cellular membrane localization (Fig. 3). Furthermore, the in vitro findings in our study underscored that PRMT6’s mediation of tumor metastasis hinged on STAT3 phosphorylation (Fig. 4). A noteworthy attribute of PRMT6 is its role as an arginine histone methyltransferase, and it extends this function to non-histone proteins as well, including but not limited to POLB, CRTC2, SIRT7, and RCC1. Notably, increasing evidence has illuminated that methylation of non-histone proteins, catalyzed by PRMT6, bears profound implications on their cellular localization, activation, stability, and interactions with other proteins [26]. However, the scenario surrounding STAT3’s arginine methylation has been somewhat contentious in the literature. While some studies have cast doubts on the presence of arginine methylation on STAT3, others have provided corroborative evidence that enzymes like PRMT1 and PRMT2 can indeed catalyze STAT3 methylation, thereby governing its transcriptional activation [33–35]. In this study, we had maken an innovative stride by substantiating that PRMT6 could asymmetrically di-methylate STAT3 at the arginine 729 site (STAT3 R729me2a). This novel form of methylation, STAT3 R729me2a, emerged as a critical factor governing the interaction between JAK2 and STAT3, the cellular membrane localization of STAT3, and its consequential phosphorylation at Y705. Our in vivo experiments provided compelling evidence that deficiency in STAT3 R729me2a significantly curbed the metastatic potential of BRCA cells, thereby reinforcing the dependence of PRMT6-mediated tumor metastasis on STAT3 R729me2a. Importantly, the clinical implications of our findings were underscored by the positive correlation among the expression levels of PRMT6, STAT3 R729me2a, and STAT3 Y705 phosphorylation in BRCA. Notably, patients exhibiting higher STAT3 R729me2a levels were associated with more advanced N and M stages and a reduced overall survival time. These observations highlight the potential utility of STAT3 R729me2a expression as a prognostic marker. For the management of BRCA, we introduced EPZ020411 as a pharmacological intervention to quell PRMT6-mediated STAT3 activation and dismantle H3R2-mediated epigenetic modifications. Our in vitro investigations illuminated the potent capability of EPZ020411 to effectively extinguish IL-6/STAT3 signaling activation and H3R2me2a levels. Additionally, it could orchestrate the reversal of epithelial–mesenchymal transition in BRCA cells. Our in vivo xenograft experiments further accentuated the promise of EPZ020411. Administering this compound significantly repressed STAT3 R729me2a, STAT3 Y705 phosphorylation, cancer cell metastasis, and ultimately extended the overall survival of mice harboring MDA-MB-468 cancer cell xenografts. These collective findings underscored the potential of PRMT6 as a viable therapeutic target in the context of STAT3-driven tumor metastasis.

## Conclusion

In summary, our study provided compelling evidence that PRMT6 functioned as an oncogene in the context of BRCA, exerting a positive regulatory influence on BRCA metastasis. Our mechanistic insights had uncovered the novel phenomenon of asymmetric di-methylation of STAT3 at arginine 729 (STAT3 R729me2a) catalyzed by PRMT6. This modification was revealed to be pivotal for various key aspects of STAT3 signaling, including its cellular membrane localization, interaction with JAK2, Y705 phosphorylation, and ultimately, PRMT6-mediated tumor metastasis. The clinical significance of our work was underscored by the identification of STAT3 as a predictive marker for assessing the overall survival of BRCA patients. As a therapeutic avenue, our findings highlighted the potential of EPZ020411 as a promising agent for mitigating BRCA metastasis, by concurrently targeting the H3R2-mediated epigenetic pathway and the IL-6/STAT3 oncogenic pathway (Fig. 8I). These insights contributed to a deeper understanding of the molecular underpinnings of BRCA metastasis and offer novel perspectives for its management and treatment.

### Supplementary information

Supplementary figure legend

Reproducibility checklist

Figure S1

Figure S2

Figure S3

Figure S4

Figure S5

Figure S6

Supplementary table 1

Original Data File

## Data Availability

All the data of this article are available.
