# Supplementary material for: PRMT6 methylation of STAT3 regulates tumor metastasis in breast cancer
Source: Cell Death Dis. 2023 Oct 9;14(10):655. doi: 10.1038/s41419-023-06148-6 (PMC10562413; doi:10.1038/s41419-023-06148-6)
Supplement: Supplementary file 1 — Supplementary figure legend [file 41419_2023_6148_MOESM1_ESM.docx]

**Supplementary Figure legend**

**Supplementary Figure 1**. **PRMT6 acted as an oncogene**. **A**. Analysis of the expression level of PRMT6 in various cancers, upper for unpaired cancer tissues, lower for paired cancer tissues. **B**. Kaplan-Meier Plot was drawn in various cancer patients grouped by the expression level of PRMT6.

**Supplementary Figure 2**. **PRMT6 overexpression upregulated the migration, invasion, and colony formation ability of cancer cells**. **A**. Immunoblotting assays confirming the successful construction of cancer cells stably expressing Vector or PRMT6. **B**. Representative image of wound healing assays of cancer cells stably expressing Vector or PRMT6. **C**. Statistical analysis for wound healing assays in Supplementary Figure 2B; n = 3, P<0.05. **D**. Representative image of transwell assays of cancer cells stably expressing Vector or PRMT6. **E**. Statistical analysis for transwell assays in Supplementary Figure 2D; n = 3, P<0.05. **F**. Representative image of colony formation assays of cancer cells stably expressing Vector or PRMT6. **G**. Statistical analysis for colony formation assays in Supplementary Figure 2**F**; n = 3, P<0.05. **H**. Representative images of MDA-MB-468 cells expressing sgRNA-control, sgRNA-PRMT6#1, and sgRNA-PRMT6#2. **I**. IHC assays to investigate the methylation and phosphorylation level of STAT3 in *in vivo* xenograft. All immunoblotting assays were conducted three times, and the results were the same.

**Supplementary Figure 3**. **PRMT6 positively regulated the IL-6/STAT3 signaling pathway**. **A**. GSEA analysis of GSE42568 and GSE2741 datasets according to the expression level of PRMT6 in breast cancer tissues. **B**. RT-PCR assays to investigate the mRNA expression level of markers of epithelial-mesenchymal transition in cancer cells stably expressing Vector or PRMT6; n = 3, P<0.05.

**Supplementary Figure 4**. **PRMT6-mediated tumor metastasis relied on STAT3 Y705 phosphorylation**. **A**, **C**. Representative image of transwell assays of cancer cells stably expressing Vector, PRMT6 with or without stattic. **B**, **D**. Statistical analysis of transwell assays shown in Supplementary Figure 4A and 4C; n = 3, P<0.05.

**Supplementary Figure 5**. **PRMT6 interacted with STAT3 and asymmetrically di-methylated STAT3 at R729**. **A**. The potential interaction between PRMT6 and STAT3 in the String database. **B**. Immunoblotting and immunoprecipitation assays to detect the targeting proteins expression level in cancer cells stably expressing Vector, PRMT6 WT, or PRMT6 KLA mutant. **C**. Immunoblotting and immunoprecipitation assays to detect the targeting proteins expression level in cancer cells with or without EPZ (20 µM) application. **D**. Co-transfecting HA-tagged STAT3 WT or HA-tagged STAT3 R729K mutant and Flag-tagged PRMT6 into HEK293T cells, immunoblotting and immunoprecipitation assays to detect the ADMA expression level of STAT3 in these four kinds of cells. **E**. Immunoblotting assays to validate the successful construction of STAT3 knock-out MDA-MB-468 cancer cells. **F**. Immunoblotting to detect the STAT3 Y705 phosphorylation expression level in MDA-MB-468 cancer cells stably expressing STAT3 WT or STAT3 R729K mutant. **G**. Immunoblotting to detect the STAT3 R729 asymmetric di-methylation expression level in cancer cells stably expressing Vector, PRMT6 WT or PRMT6 KLA mutant. **H**. Immunoblotting to detect the STAT3 R729 asymmetric di-methylation expression level in MDA-MB-468 cancer cells with or without EPZ. **I**. Representative image of IHC assays using PRMT6, P-STAT3, or STAT3 R729 antibody. **J**. Scatter plot showing the expression level of STAT3 phosphorylation (left) or STAT3 R729 methylation (right) and PRMT6; n = 62, P<0.05. **K**. Statistical analysis of the expression level of STAT3 R729 methylation in breast cancer patients grouped by N or clinical stage; n = 62, P<0.05. All immunoblotting assays were conducted three times, and the results were the same.

**Supplementary Figure 6**. **Targeting PRMT6 might be a work-well method for breast cancer treatment**. **A**. Representative image of transwell assays in cancer cells stably expressing STAT3 WT + vector, STAT3 WT + PRMT6, STAT3 R729K + vector, and STAT3 R729K + PRMT6. **B**, **C**. Representative image of transwell assays in cancer cells with or without EPZ (20 µM).
