# Supplementary figures and images for: PRMT6 methylation of STAT3 regulates tumor metastasis in breast cancer
Source: Cell Death Dis. 2023 Oct 9;14(10):655. doi: 10.1038/s41419-023-06148-6 (PMC10562413; doi:10.1038/s41419-023-06148-6)

Figure 1

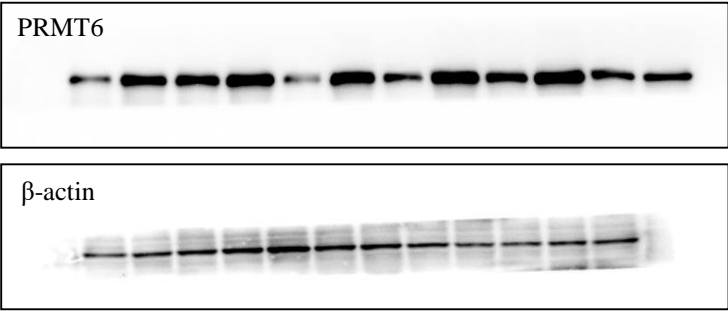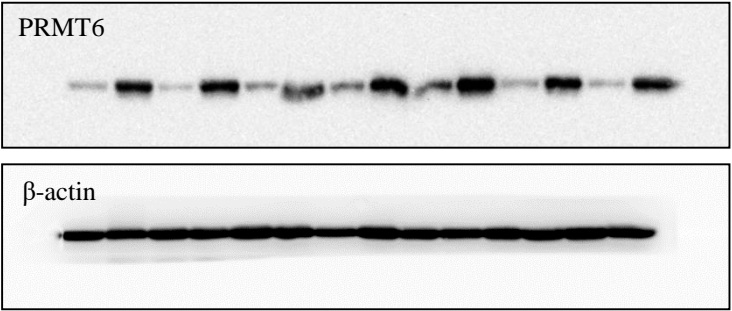

Figure 2

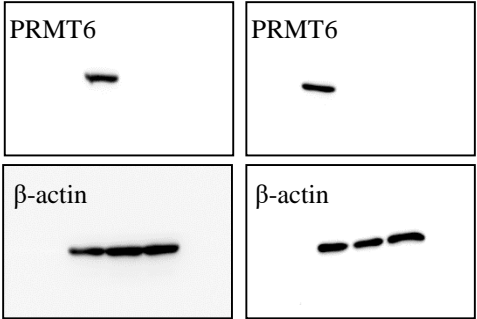

Figure 3

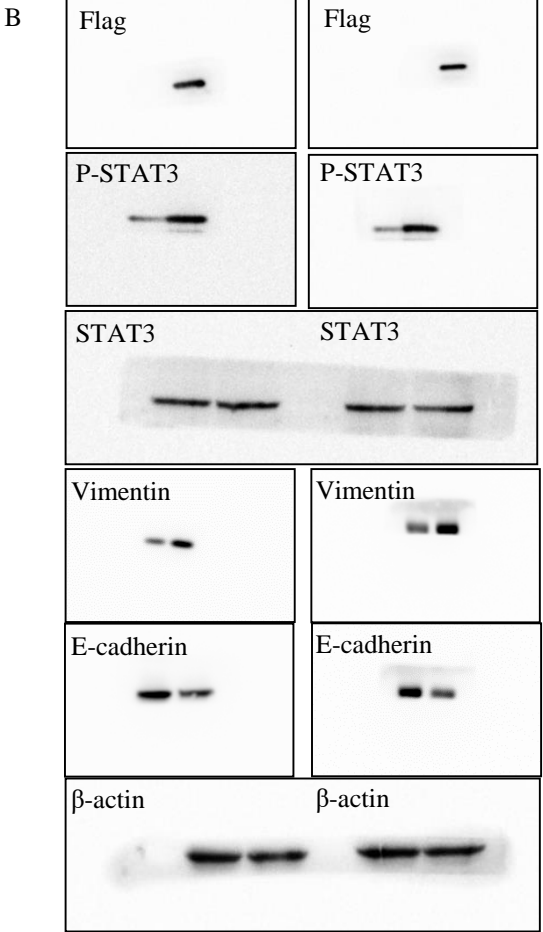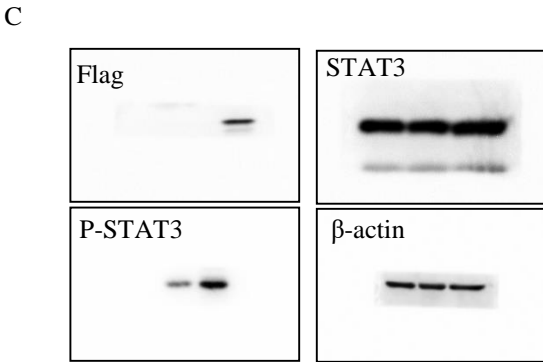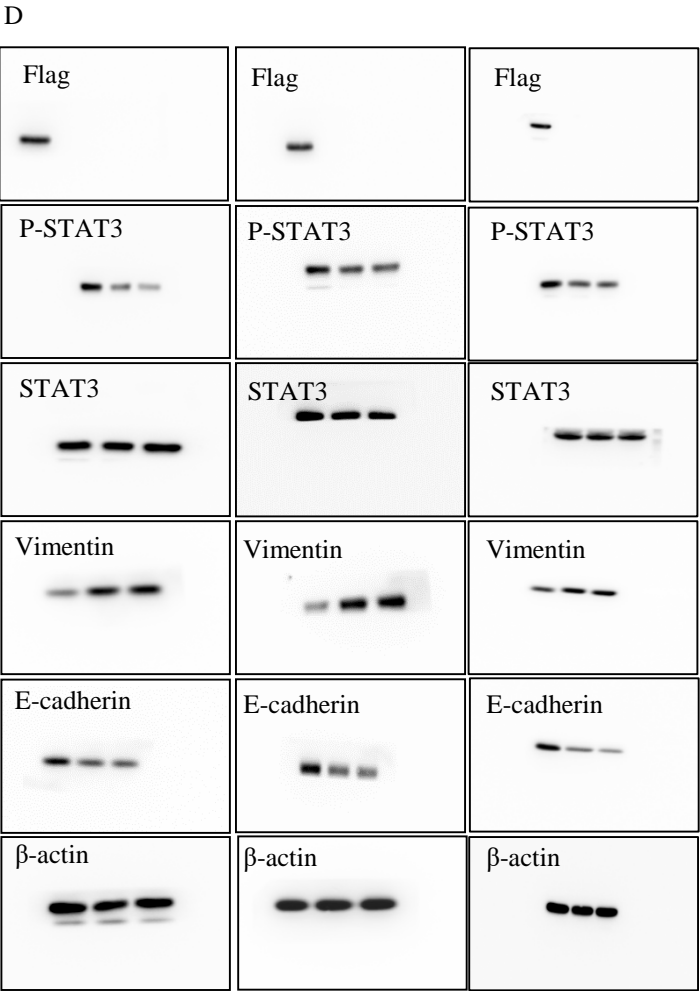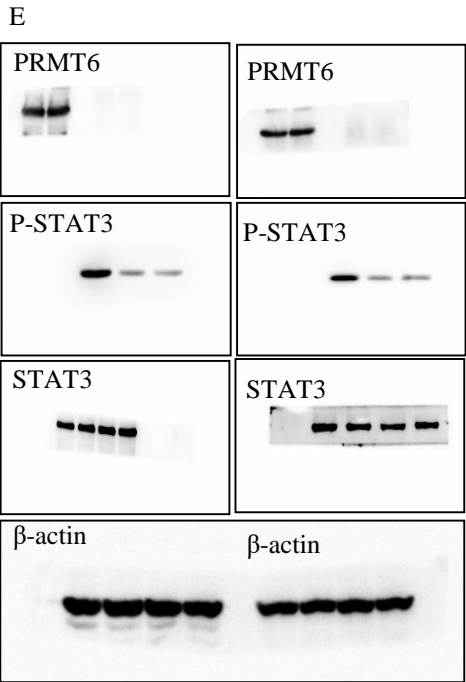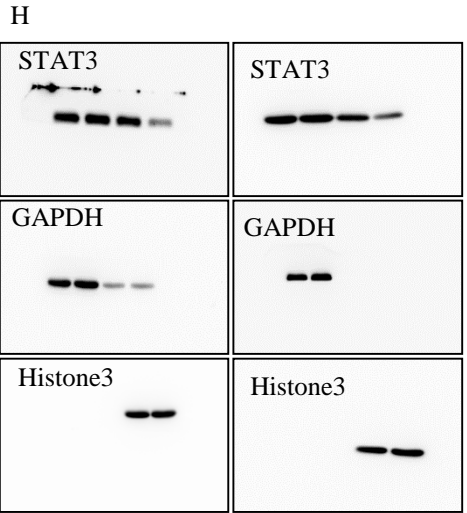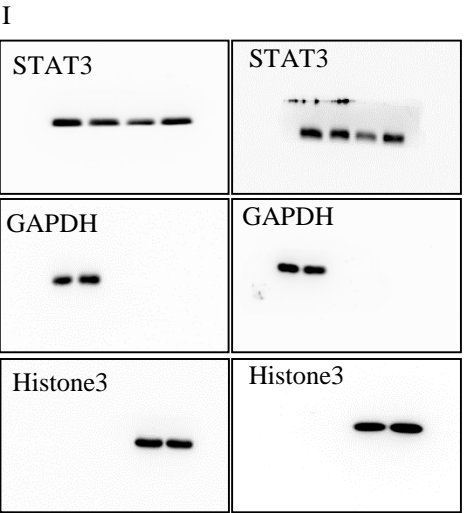

Figure 4

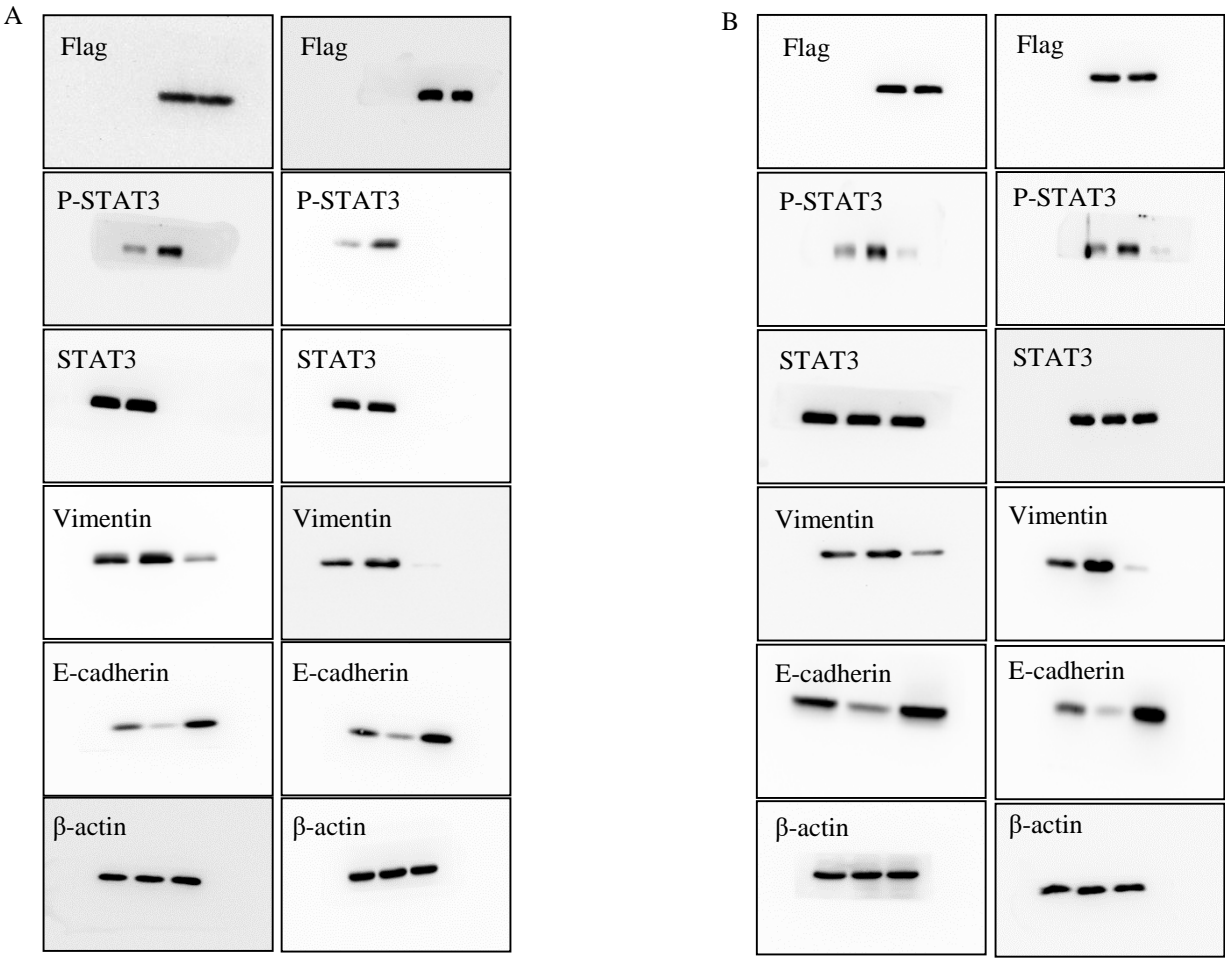

Figure 5

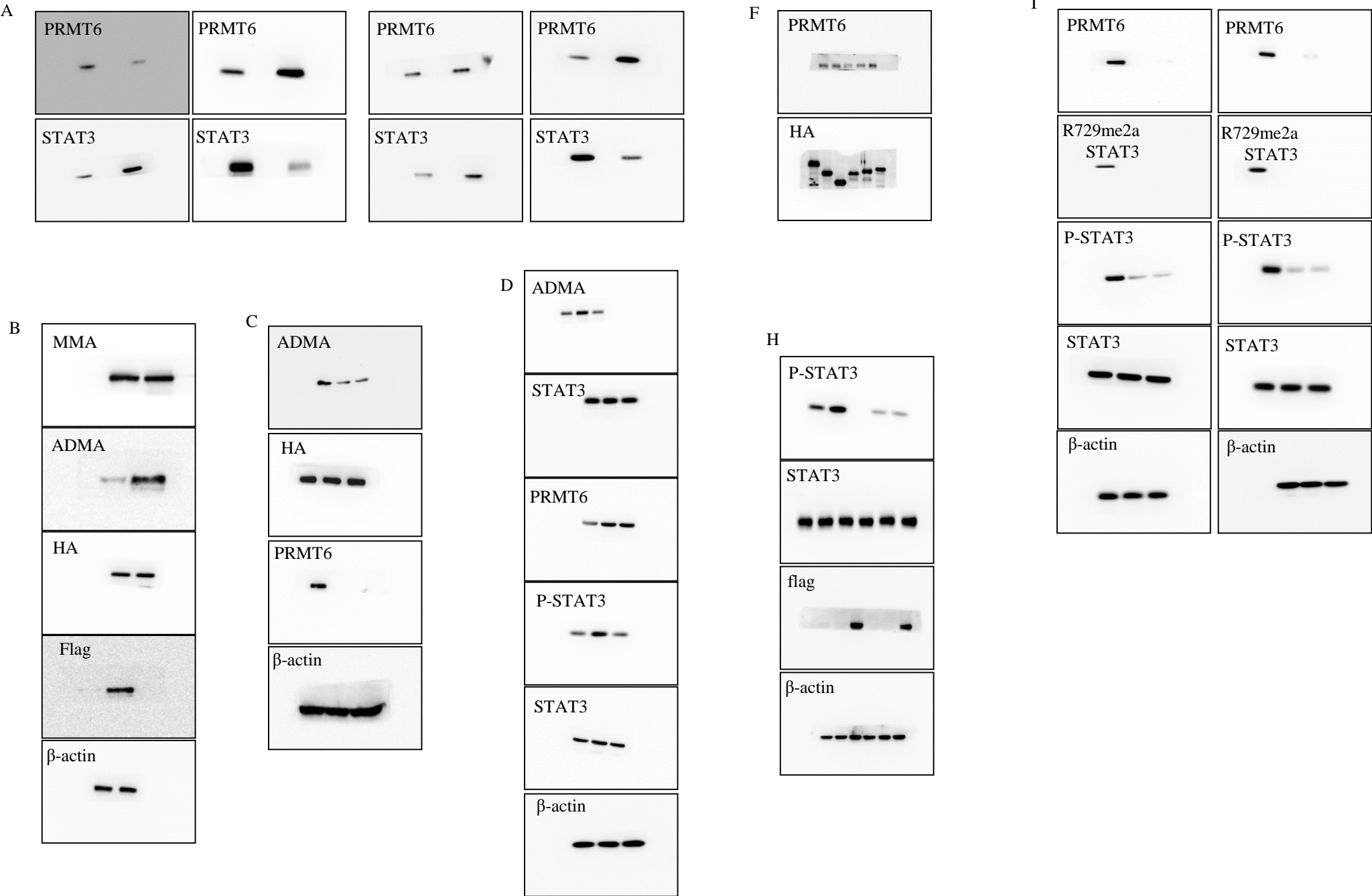

Figure 6

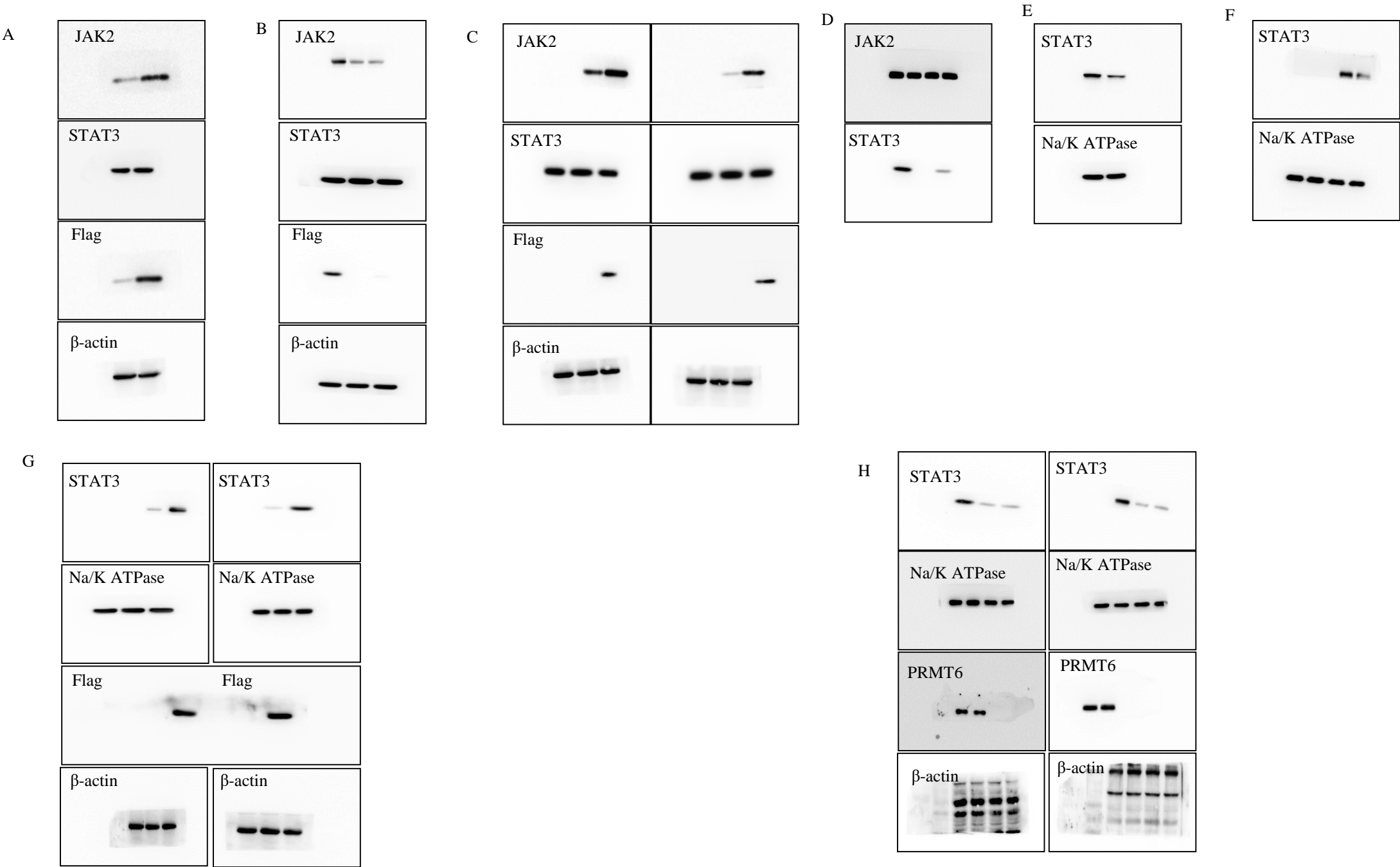

Figure 7

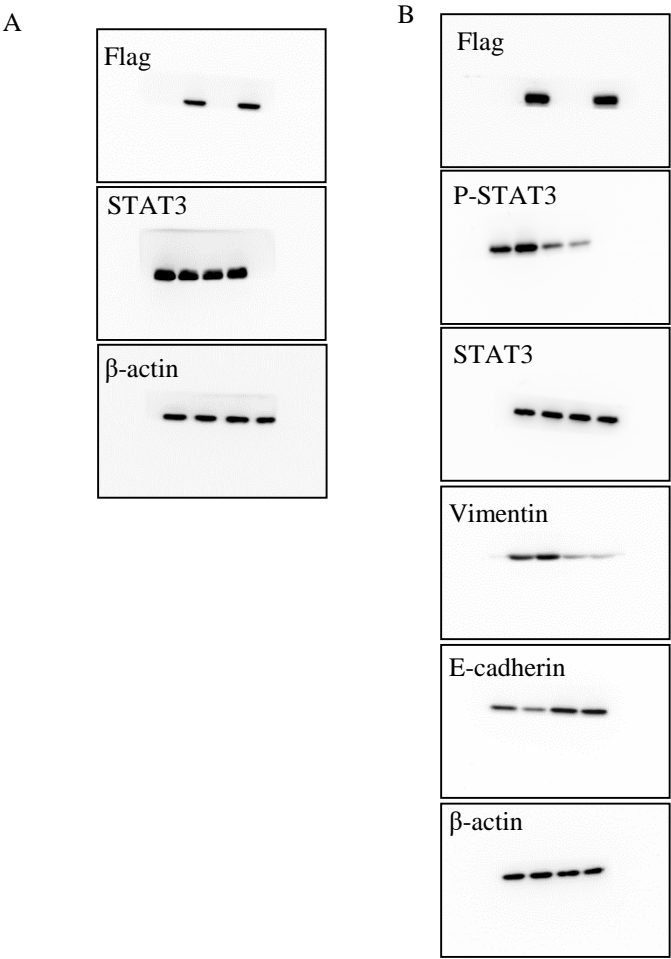

Figure 8

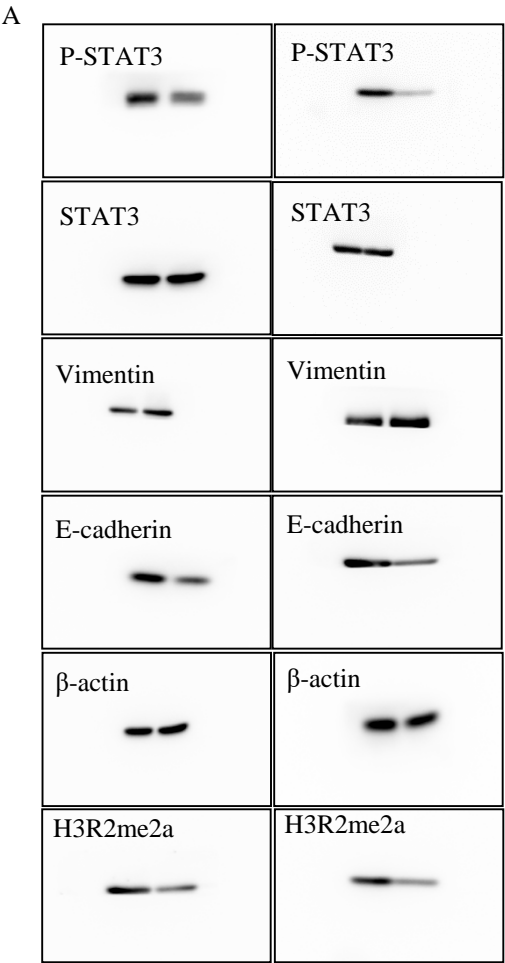

Figure S2

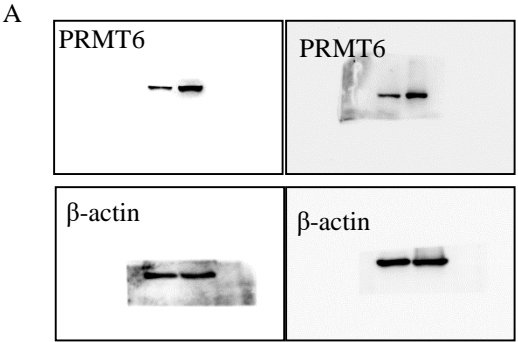

Figure S5

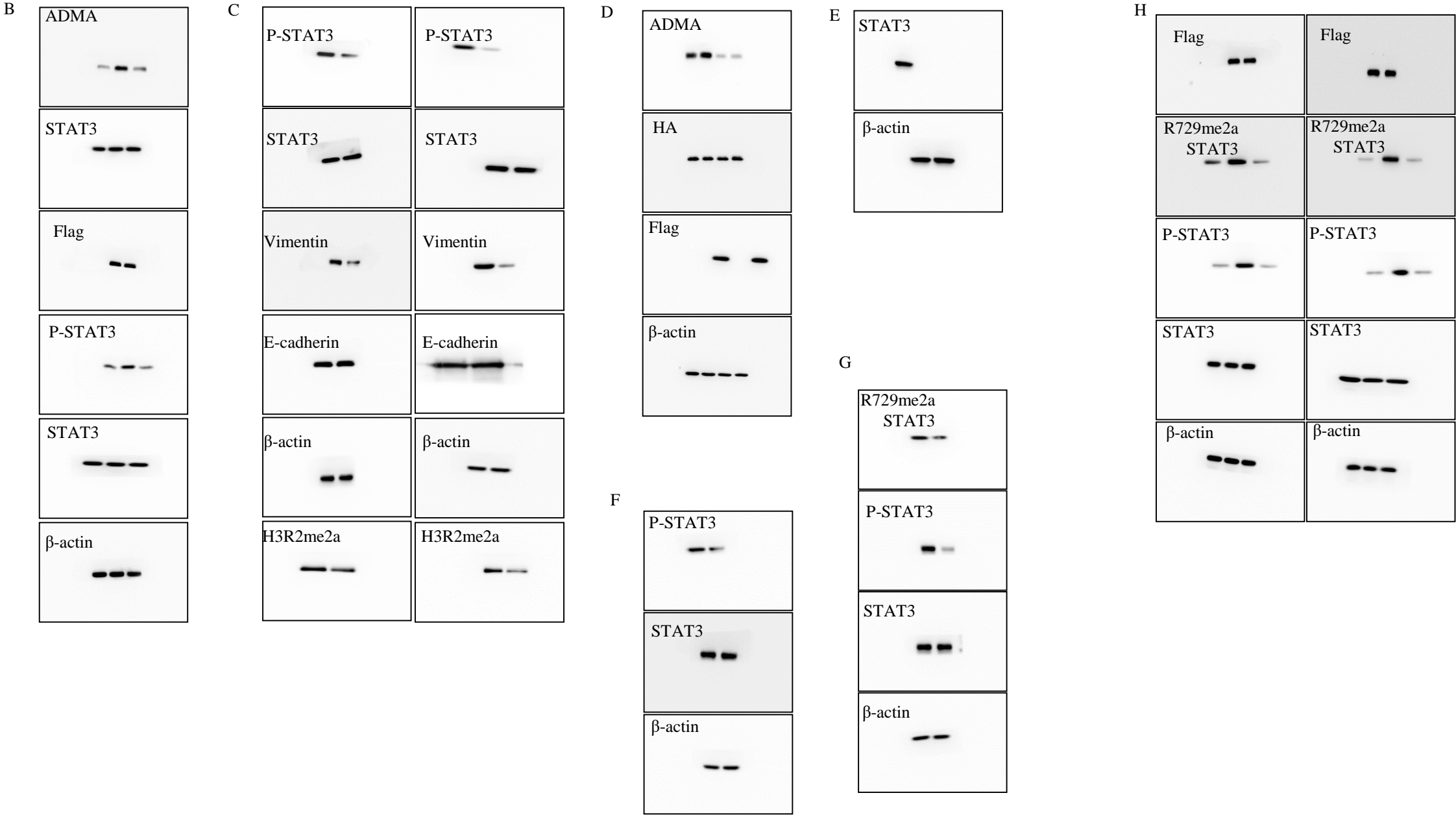

Supplement: Supplementary file 10 — Original Data File [file 41419_2023_6148_MOESM10_ESM.pdf]
